# Colistin: recent data on pharmacodynamics properties and clinical efficacy in critically ill patients

**DOI:** 10.1186/2110-5820-1-30

**Published:** 2011-08-02

**Authors:** Argyris S Michalopoulos, Matthew E Falagas

**Affiliations:** 1 Department of Critical Care Medicine, Henry Dunant Hospital, Mesogeion 107, 11526 Athens, Greece; 2 Alfa Institute of Biomedical Sciences (AIBS), 9 Neapoleos Street, 151 23 Marousi, Greece; 3 Department of Medicine, Henry Dunant Hospital, Mesogeion 107, 11526 Athens, Greece; 4 Department of Medicine, Tufts University School of Medicine, Boston, MA, USA

## Abstract

Recent clinical studies performed in a large number of patients showed that colistin "forgotten" for several decades revived for the management of infections due to multidrug-resistant (MDR) Gram-negative bacteria (GNB) and had acceptable effectiveness and considerably less toxicity than that reported in older publications. Colistin is a rapidly bactericidal antimicrobial agent that possesses a significant postantibiotic effect against MDR Gram-negative pathogens, such as *Pseudomonas aeruginosa*, *Acinetobacter baumannii*, and *Klebsiella pneumoniae*. The optimal colistin dosing regimen against MDR GNB is still unknown in the intensive care unit (ICU) setting. A better understanding of the pharmacokinetic-pharmacodynamic relationship of colistin is urgently needed to determine the optimal dosing regimen. Although pharmacokinetic and pharmacodynamic data in ICU patients are scarce, recent evidence shows that the pharmacokinetics/pharmacodynamics of colistimethate sodium and colistin in critically ill patients differ from those previously found in other groups, such as cystic fibrosis patients. The AUC:MIC ratio has been found to be the parameter best associated with colistin efficacy. To maximize the AUC:MIC ratio, higher doses of colistimethate sodium and alterations in the dosing intervals may be warranted in the ICU setting. In addition, the development of colistin resistance has been linked to inadequate colistin dosing. This enforces the importance of colistin dose optimization in critically ill patients. Although higher colistin doses seem to be beneficial, the lack of colistin pharmacokinetic-pharmacodynamic data results in difficulty for the optimization of daily colistin dose. In conclusion, although colistin seems to be a very reliable alternative for the management of life-threatening nosocomial infections due to MDR GNB, it should be emphasized that there is a lack of guidelines regarding the ideal management of these infections and the appropriate colistin doses in critically ill patients with and without multiple organ failure.

## Colistin's pharmacodynamic properties

Colistimethate sodium (CMS) is an inactive prodrug of colistin that exhibits a low level of protein binding. It is not stable *in vitro *and *in vivo *and is hydrolyzed in human plasma, creating a complex mixture of partially sulphomethylated derivatives with the potential to produce up to 32 different products, including colistin [1]. After administration of CMS, colistin appears in plasma rapidly. Colistin is approximately 50% bound to human plasma. Peak serum levels after intravenous (i.v.) administration are achieved within 10 min. They appeared higher but declined more rapidly than those achieved after i.m. administration [2].

Colistin (base) is more active than CMS. Serum half-life of CMS is approximately 1.5-2 hours (h) after i.v. administration and 2.75 to 3 h after i.m. administration in healthy subjects, whereas serum half-life for CMS administered i.v. is more than 4 h. Old reports have suggested that colistin is poorly distributed to the pleural cavity, lung parenchyma, bones, and cerebrospinal fluid (CSF) (15% to 25%).

CMS is eliminated predominantly by the kidneys. It should be noted that after CMS i.v. administration, approximately 60% of CMS is excreted unchanged in the urine via glomerular filtration during the first 24 h. In renal failure, the renal excretion of CMS is decreased resulting in a higher conversion to colistin and prolongation of half-life [3]. On the contrary, colistin is eliminated predominantly by the nonrenal route by means of mechanisms not yet fully understood [4]. However, in humans, colistin is not absorbed from the gastrointestinal tract and no biliary excretion has been reported.

The pharmacodynamic (PD) properties of colistin, such as minimal inhibitory concentration (MIC), mutation prevention concentration, population analysis profile, bacterial-killing kinetics, and the postantibiotic effect (PAE) against multidrug-resistant (MDR) Gram-negative bacteria (GNB), such as *Pseudomonas aeruginosa*, *Acinetobacter baumannii*, and *Klebsiella pneumoniae*, have been examined in recent studies [5,6]. Based on the study by Owen et al. [5] colistin seems to be very active in the initial killing of *A. baumannii*, even with 0.5 × MIC, exhibiting a concentration-dependent bacterial-killing mechanism. Modest positive PAEs of colistin were observed at relatively high concentrations (≥16 × MIC), which are not achieved in clinical practice. The most significant finding of the study was the substantial regrowth occurring at 24 h even at colistin concentrations up to 64 × MIC and the minor or negative PAE of colistin [7]. These findings were consistent with the hetero-resistance of *A. baumannii *isolates to colistin, reported in previous studies, suggesting that CMS monotherapy and extended-interval dosage regimens may be problematic for the effective treatment of nosocomial infections caused by colistin-heteroresistant *A. baumannii *in the intensive care unit (ICU) setting [7].

Poudyal et al. [6] found initial rapid killing against *K. pneumoniae *strains even at the lowest colistin concentration. Similarly with the previous study dealing with the colistin pharmacodynamics against MDR *A. baumannii*, colistin exhibited no PAE at up to 64 × MIC, regrowth in the majority of isolates at 4 h and hetero-resistance to colistin in MDR but colistin-susceptible *K. pneumoniae *strains. These findings suggest that CMS monotherapy and extended-interval dosage regimens, as has been aforementioned for *A. baumannii *isolates, may promote colistin resistance in MDR *K. pneumoniae *strains

## Colistin's pharmacokinetic properties

The main pharmacokinetics (PK) of colistin are presented in Table 1. Few studies have addressed the PKs of CMS and colistin in humans, especially in the ICU setting. It should be emphasized that significant pharmacodynamic parameters, such as Cmax/MIC ratio, AUC/MIC, and T > MIC that could best predict colistin efficacy and safety have not yet been clearly defined in humans in critically ill patients. For this reason, the optimum target for colistin Cmax/MIC ratio is not yet known. In addition, there is still a lack of PK/PD information to optimize colistin doses in humans, especially those who are hospitalized in the ICUs. A better understanding of the CMS and colistin (base) PKs could be beneficial for colistin use in humans. It is known that CMS and colistin (base) PKs differ, given that they have different structures, antibacterial activity, and toxicity.

**Table 1:** Pharmacokinetics of colistin (CMS)

Metabolism: CMS is a prodrug that is hydrolyzed after i.v. administration to produce derivatives, including the active drug colistin
It is not absorbed from the gastrointestinal tract
Distribution of CMS to lung parenchyma, pleural cavity, pericardial fluids, and CSF is poor
Time to peak: 10 min following i.v. administration
Half-life elimination: 2-3 h (CMS i.v. administration, with normal renal function). In patients with anuria = 2-3 days.
For colistin (base): 250 min
CMS is tightly bound to membrane lipids of cells in many body tissues, including liver, lungs, kidneys, brain, heart, and muscles
CMS is excreted primarily in the urine (as unchanged drug). No biliary excretion has been reported in humans
Data on the pharmacokinetics of i.v. CMS in critically ill patients are limited

Bergen et al. [8] examined the pharmacokinetics of colistin in an *in vitro *pharmacokinetic/pharmacodynamic model. Three intermittent dosage regimens involving 8-h, 12-h, and 24-h dosage intervals (Cmax of 3.0, 4.5, or 9.0 mg/L, respectively) were administered in humans. Antibacterial activity and emergence of resistance were investigated during the 72-hour treatment period using two strains of *P. aeruginosa*. No difference in overall bacterial killing was found. However, the 8-hourly regimens appeared most effective at minimizing the onset of resistance. This study additionally showed that the AUC:MIC ratio of total and unbound colistin is the index that best predicts the antibacterial activity against *P. aeruginosa*, superior to Cmax/MIC, suggesting that time-averaged exposure to colistin is more important than the achievement of high peak concentrations. The PK/PD relationship of colistin against *P. aeruginosa *has been examined recently in a vitro model. A significant finding of the study was that colistin efficacy against *P. aeruginosa *was best correlated with the AUC:MIC ratio of total and unbound colistin rather than the Cmax/MIC ratio. As a consequence, the time-averaged exposure to colistin is a more important target in the clinical practice than the achievement of high colistin peak concentrations [9].

Steady-state pharmacokinetics of colistin has been recently examined in 13 adult patients with ventilator-associated pneumonia (VAP) caused by GNB. Patients were treated with CMS: 2 million (m.) units that are equivalent with 174 mg CMS, administered i.v. every 8 hours, for at least 2 days. Patients received a mean of 2.19 mg/kg of CMS per dose. At steady-state, apparent volume of distribution (Vd/fm) was 1.5 ± 1.1 L/kg. Cmax/MIC ratio and AUC0-24/MIC ratio (for MIC = 2 mcg/ml) were 1.1 ± 0.5 and 17.3 ± 9.3, respectively. The authors also examined the colistin concentration in BAL, which was found to be undetectable. Based on these findings, it seems that the reported daily colistin dose of 6 m. units (2 m. units administered every 8 hours) resulted in suboptimal serum colistin concentrations and undetectable colistin concentrations in BAL in critically ill patients [10].

It should be noted that recently Dudhani et al. used two murine infection models to identify the most predictive PK/PD index of the antibacterial activity of colistin against *P. aeruginosa *and *A. baumannii strains*. The authors reported that fAUC/MIC was the most predictive PK/PD index that correlated best with colistin efficacy against these Gram-negative pathogens in both thigh and lung infection models. These studies highlighted the importance of achieving adequate time-averaged exposure to colistin across the day [11,12]. These studies performed in animals will facilitate efforts to define in the near future the more rational design of CMS doses in humans, especially in the ICU setting.

## Optimizing colistin dose based on PK/PD properties

The optimal dose of colistin has not been determined in the ICU setting, because since there is a lack of relative clinical studies. In addition, there is lack of colistin's PK/PD data in critically ill patients. Reliable colistin PK/PD data, a better understanding of these data, and recent randomized, controlled trials are necessary to redefine appropriate colistin doses. This strategy relates to all potential routes of colistin administration to maximize colistin clinical efficacy associated with minimal adverse effects. In addition, there is discrepancy regarding the recommended doses of colistin (CMS) worldwide. This fact is mainly based on two major parameters: 1) the amount of colistin included in each vial of colistin in different countries is different; and 2) some vials refer to CMS but others in colistin base. It seems that the best way to avoid confusion related to colistin dosing is to base the doses on international units. Pure colistin base has been assigned a potency of 30,000 IU per mg, and CMS has a potency of 12,500 IU per mg. Thus, recommendations regarding dosing of colistin should clearly refer to colistin base or colistimethate sodium to avoid possible confusion. The recommended doses of CMS in patients with normal renal function, those with renal failure, and those who undergo renal replacement therapy or peritoneal dialysis are presented in Table 2.

**Table 2:** Recommended doses of i.v. colistin (CMS) in critically ill patients

Normal renal function
3 million IU (240 mg CMS) every 8 h
Manufacturers of European colistin products recommend 50,000 to 75,000 IU/kg/day of CMS in 2-3 divided doses
Manufacturers of the U.S. colistin product, Coly-Mycin, recommend a dose of 2.5 to 5 mg/kg colistin base activity daily divided in 2 to 4 doses
Renal Failure
For serum creatinine level 1.3-1.5 mg/dl, 1.6-2.5 mg/dl, or ≥ 2.6 mg/dl, the recommended dosage of intravenous colistin is 2 million IU (160 mg CMS) every 8 h, 12 h, or 24 h, respectively
Renal replacement therapy
2 million IU (160 mg CMS) after each hemodialysis
2 million IU (160 mg CMS) daily during peritoneal dialysis

The steady-state colistin serum concentrations have been measured in 14 septic patients with stable renal function in a general ICU after i.v. administration of CMS. The CMS dose was 225 mg administered every 8 or 12 h for at least 2 days. At steady state, mean maximum and minimum colistin concentrations were 2.93 and 1.03 mg/L, respectively, whereas mean apparent total body clearance was 13.6 L/h, apparent volume of distribution was 139.9 L, and t(1/2) was 7.4 h. Cmax/MIC ratio displayed a wide range of values. The authors reported that with colistin sensitivity defined as a MIC break point ≤2 μg/mL, the Cmax levels achieved with this colistin dose would most probably lead to suboptimal Cmax/MIC ratios for many isolated strains in the upper range of these MIC values. The authors concluded that higher doses of CMS should be considered in critically ill patients [13].

CMS and colistin PKs have been recently examined in 18 adult critically ill patients who received i.v. colistin for infections caused by MDR-GNB. CMS was administered at a dose of 3 m. units (240 mg) every 8 h (or 160 mg every 8 h if creatinine clearance was < 50 ml/min). The clearance of CMS was 13.7 L/h. For colistin, the estimated half-life was 14.4 h. The predicted maximum concentrations of drug in plasma were 0.60 mg/L for the first dose and 2.3 mg/L at steady state. After the first few doses, colistin concentrations were below the Clinical and Laboratory Standards Institute MIC breakpoint of 2 mg/L for *P. aeruginosa *and Enterobacteriaceae. In addition, at steady state, plasma concentrations were below the MIC breakpoints for many of the cases. At daily colistin doses of 9 m. units (3 m. units administered every 8 h), it would take 2-3 days before the steady-state concentration was achieved. A significant finding of the study was that colistin displayed a significantly longer half-life in relation to the dosing interval. The authors speculated that a loading colistin dose of 9 or 12 m. units along with a maintenance dose of 4.5 m. units administered every 12 h is necessary in critically ill patients [14].

Another important aspect to be determined is the colistin frequency of dosing in critically ill patients. The PKs of three different CMS daily doses (3 m. units every 8 h 4.5 m. units every 12 h and 9 m. units every 24 h) have been recently examined by Daikos et al. [15]. The authors evaluated the bactericidal activity of serum containing various concentrations of colistin against *P. aeruginosa *with a MIC 1 μg/ml. Mean serum C (max) of colistin were 3.34, 2.98, and 5.63 μg/ml, respectively. All serum samples containing colistin > 4 μg/ml (serum colistin concentration/MIC > 4) eliminated *P. aeruginosa*, whereas only 40% of samples containing colistin < 4 μg/ml resulted in complete bacterial killing. Based on these findings, the currently used colistin dosing regimens might not provide the most effective therapy and therefore might justify administering larger colistin doses in longer intervals. However, although the potential for a longer dosing interval may be an option in critically ill patients, some studies found that when the intervals of colistin doses increase, the prevalence of colistin resistance also increases.

Clearance of CMS and colistin was found to be lower, whereas conversion of CMS to colistin and overall colistin exposure were increased in patients with renal failure compared with healthy subjects. No clinical data exist on colistin dosing for patients receiving continuous renal replacement therapy. Based on the PK properties of colistin, the recommended dose of colistin in this group of patients is 2.5 mg/kg q 48 h. However, there are serious doubts about this recommendation. It is likely that higher colistin dosage (e.g., 2 to 3 mg/kg every 12 h) is necessary. In a patient undergoing continuous venovenous hemodiafiltration, conversion of CMS to colistin was rapid, and the terminal half-lives of CMS and colistin were 6.8 and 7.5 h, respectively [16]. Based on older studies, in patients with renal failure undergoing peritoneal dialysis, approximately 1 mg/h of colistin is removed from the patient and approximately 16% of the total colistin dose is removed during a 2-h peritoneal dialysis session. Because of this poor clearance, the recommended dose of colistin should be 2 mg/kg/day.

During the past decade, inhaled colistin has been used for the treatment of nosocomial pneumonia or VAP due to MDR GNB, mostly *P. aeruginosa *and *A. baumannii*, to improve lung parenchyma penetration. Although administration of colistin via inhalation has been adopted and recommended to improve lung parenchyma penetration in the adjunct treatment of MDR pneumonia or VAP, there are until now few data on the PKs of colistin after inhalation. In addition, no study has been performed to assess the colistin concentrations achieved in the pulmonary epithelial lining fluid, which is the target site for antibiotics, in the treatment of pneumonia. The first study that evaluated the colistin pharmacokinetics postinhalation was conducted by Ratjen et al. [17] in patients with cystic fibrosis. In this multicenter study, a single dose of CMS (2 m. units) was administered via inhalation to assess sputum, serum, and urine concentrations. An interesting finding of this study was that the maximum sputum concentrations of colistin were at least 10 times higher than those proposed by the British Society for Antimicrobial Chemotherapy. Although sputum drug concentrations decreased after a peak at 1 h, the mean colistin concentrations remained above 4 mg/L after 12 h. Serum concentrations of colistin reached their maximum at 1.5 h after inhalation and decreased thereafter. A mean of 4.3 ± 1.3% of the inhaled dose was detected in urine.

Lu et al. [18] compared lung tissue deposition and antibacterial efficiency between nebulized and intravenous administration of colistin in piglets with pneumonia caused by *P. aeruginosa*. CMS was administered either by nebulization every 12 h or i.v. every 8 h. The fraction of CMS reaching the respiratory tract was 60% of the initial dose. An interesting finding of this study was that colistin was undetected in lung tissue after intravenous infusion. On the contrary, median colistin peak lung concentration after nebulization was 2.8 μg/g. After three consecutive CMS aerosols, peak tissue concentrations were found higher than MIC, indicating significant distal lung deposition. In the aerosol group of piglets, peak lung tissue concentrations were significantly higher in lung segments with mild pneumonia (median = 10.0 μg/g) compared with lung segments with severe pneumonia (median = 1.2 μg/g; *p *< 0.01). After 24 h of colistin treatment, 67% of pulmonary segments had bacterial counts < 10^2 ^cfu/g after nebulization and 28% after i.v. administration (*p *< 0.001). On the contrary, in control animals, 12% of lung segments had bacterial counts < 10^2 ^cfu/g 49 h after bronchial inoculation.

Although these data seem promising, it is not known whether they can be extrapolated to critically ill patients with MDR nosocomial pneumonia in the ICU setting, who may display different pharmacokinetics parameters compared with patients with cystic fibrosis. Hence, pharmacokinetic data regarding inhaled colistin in ICU patients with MDR VAP are very much warranted.

Only a few case reports in the literature deal with the intrathecal administration of colistin for the treatment of ventriculitis and shunt infections due to carbapenem resistant *P. aeruginosa *and *A. baumannii *[19-21]. Markantonis et al. [22] examined recently colistin concentrations in serum and CSF samples in five critically ill patients who received CMS for CNS infections due to MDR GNB. The objective of this study was to investigate colistin's penetration into the CSF. However, they found low penetration level (5%) suggesting inadequate bactericidal colistin concentrations in the CSF.

The treatment of postneurosurgical meningitis or ventriculitis or CNS shunt infection due to MDR GNB, such as *A. baumannii *and *P. aeruginosa*, is a difficult clinical problem and is associated with high mortality rates mainly due to the limited penetration of colistin into the CSF. There are few case reports dealing with the successful management of postneurosurgical ventriculitis due to MDR *A. baumannii *or *P. aeruginosa strains *treated successfully and safely with CMS administered by the intrathecal or intraventricular route. The dosage of colistin (base) for intraventricular administration ranges from 5 mg to 20 mg per day (divided in 1 or 2 doses). In our patients, we administer 500,000 IU CMS once per day intraventricularly or directly into CSF for 2 consecutive days followed by 300,000 IU once per day for the following 5-7 days. The median time necessary to obtain CSF sterilization seems to be approximately 5 days. Toxicity probably or possibly related to the topical administration of colistin is noted in approximately 15% of patients.

## Colistin's clinical efficacy in critically ill patients

In a recently published study, 258 adult critically ill patients (mean age 61 years) received i.v. colistin for at least 72 hours for microbiologically documented MDR Gram-negative infections mainly due to *A. baumannii *(65.9%) and *P. aeruginosa *(26.4%). The mean duration of hospital and ICU stays until the start of colistin administration for the index infection was 18.3 and 11.4 days, respectively. The mean duration of colistin administration was 17.9 days and the interquartile range was 10-22 days. Cure of infection occurred in 79.1% of patients. An interesting finding of this study was that nephrotoxicity occurred in only 10% of patients [23]. Similar rates of nephrotoxicity are reported by other studies [24-26]. please, delete reference No 11 On the contrary, Koomanachai et al. and Kim et al. reported a colistin-induced nephrotoxicity in approximately 30% of patients [27,28].

Apart from adults, intravenous colistin also has been administered with safety and efficacy in children and neonates, including preterm and extremely low birth weight neonates [29-31].

## Conclusions

Numerous recent clinical studies have confirmed that colistin is an efficient antimicrobial agent against nosocomial infections, including bacteremia, ventilator-associated pneumonia, urinary tract infection, and meningitis due to MDR GNB, such as *P. aeruginosa, A. baumannii*, and *K. pneumonia*, with an acceptable safety profile. Whereas colistin is mainly administered i.v. in critically ill patients, it can be safely be administered by inhalation in patients with pneumonia/VAP or intrathecally in patients with meningitis due to MDR GNB. Although colistin PK/PD data are scarce in ICU patients, recent evidence shows that the PK/PD properties of CMS and colistin are different in critically ill patients compared with other groups, such as patients with cystic fibrosis. A better understanding of colistin PK-PD properties is urgently needed to determine the optimal dosing regimen in colistin monotherapy or combination therapy for the effective management of life-threatening nosocomial infections due to MDR GNB in critically ill patients.

## Competing interests

Argyris Michalopoulos declares that he has no competing interests. Mathew E. Falagas has participated in advisory boards of Pfizer, Astellas, and Bayer and has received lecture honoraria from Merck, Pfizer, AstraZeneca, Astellas, Cipla, Novartis, and Glenmark.

## Authors' contributions

MA wrote the first draft of the manuscript. MEF did substantial revisions. Both authors approved the final version of the manuscript.
